# Exosome-mediated modulation of macrophage polarization and inflammation in early *Klebsiella pneumoniae* lung infections

**DOI:** 10.1042/CS20256616

**Published:** 2025-12-23

**Authors:** Ziyu Liu, Ping Ren, Ying Xue, Shouyue Liu, Shanyu Li, Yanan Li, Ying Zhang

**Affiliations:** 1 Department of Pediatric Respiratory Medicine, Children’s Medical Center, The First Hospital of Jilin University, Changchun, China; 2 Department of Hepatobiliary and Pancreatic Surgery, General Surgery Center, The First Hospital of Jilin University, Changchun, China; 3 Department of Thoracic Surgery, General Surgery Center, The First Hospital of Jilin University, Changchun, China; 4 HOOKE Instruments Ltd., Changchun, China; 5 Department of Neurosurgery, The Second Hospital of Jilin University, Changchun, China

**Keywords:** exosomes, *Klebsiella pneumoniae*, macrophage polarization, NF-κB signaling, pulmonary infection

## Abstract

*Klebsiella pneumoniae* is a major pathogen responsible for severe pulmonary infections, yet the early mechanisms of infection remain incompletely understood. This study investigates the role of exosomes derived from *K. pneumoniae* in polarizing macrophages to the M1 phenotype, thereby facilitating early lung infections. Utilizing single-cell Raman spectroscopy, we rapidly detected *K. pneumoniae* within host cells and observed significant lipid expression changes. Metabolomic analysis of exosomes from infected epithelial cells uncovered an elevation of phosphatidylcholine, which disrupted endothelial tight junctions and promoted M1 macrophage recruitment and polarization. This process activated the NF-κB signaling pathway, increasing inflammatory responses and attracting neutrophils. Our findings, validated in infected tissue models, suggest that these exosomal mechanisms significantly contribute to the early stages of pulmonary infection by *K. pneumoniae*. This study offers crucial insights into potential therapeutic targets for controlling *K. pneumoniae* infections.

## Introduction

Bacterial pneumonia continues to pose a significant global health challenge, with *Klebsiella pneumoniae* emerging as a major contributor to this burden [1]. As a gram-negative bacterium, *K. pneumoniae* is an opportunistic pathogen responsible for severe infections, including pneumonia, liver abscesses, and bacteremia, particularly in both healthy and immunocompromised individuals [2]. This pathogen accounts for 5–20% of gram-negative sepsis cases [3], with mortality rates ranging from 27.4% to 37% [4]. In the United States, *K. pneumoniae* ranks as the third leading cause of hospital-acquired infections, following *Clostridium difficile* and *Staphylococcus aureus* [5], and is the primary cause of ventilator-associated pneumonia in ICU patients [6].

Exosomes are nanoscale vesicles secreted by nearly all cells, playing vital roles in intercellular communication by transferring proteins, nucleic acids, lipids, and metabolites. Recent advances in mass spectrometry and high-throughput sequencing have facilitated the large-scale characterization of exosomes [7]. Although research on exosomes in bacterial infections is limited, studies suggest that exosome-derived miRNAs can enhance immune responses in pulmonary infections, such as those caused by Pseudomonas aeruginosa. In *K. pneumoniae* infections, specific miRNAs inhibit inflammasome activation in macrophages, indicating a crucial role for exosomes in modulating immune responses [8,9].

Macrophages exhibit diverse functions and phenotypes, influenced by their microenvironment [10]. They polarize into either M1 or M2 phenotypes, with M1 macrophages being pro-inflammatory and M2 macrophages exerting immunomodulatory effects. Macrophage polarization can be categorized into two phenotypes. One phenotype is the classically activated macrophages, namely M1 macrophages, which are usually induced by Toll-like receptor (TLR) ligands. This subgroup of macrophages expresses TLR2, TLR4, CD80, CD86, inducible nitric oxide synthase (iNOS), and major histocompatibility complex II (MHCII); and has high antigen presentation ability and pro-inflammatory cytokine expression [11,12]. The other phenotype is the alternative activated macrophages, namely M2 macrophages, which express specific antigens such as CD206, CD163, CD209, FIZZ1, and Ym1/2. M2 macrophages can be further categorized into M2a, M2b, M2c, and M2d subtypes and exert immunomodulatory effects through different mechanisms [13]. Understanding how macrophage polarization occurs in different contexts is essential for comprehending disease processes [14].

Raman spectroscopy provides a unique method for identifying microorganisms by detecting inelastically scattered photons, which produce specific vibrational fingerprints of molecular bonds [15–18]. This technique offers high resolution and the ability to detect multiple analytes simultaneously, making it valuable for rapid microbial identification [19–21]. Beyond its biophysical advantages, Raman spectroscopy has been widely applied in microbial diagnostics. It enables label-free and culture-independent detection by capturing the biochemical fingerprints of nucleic acids, proteins, lipids, and cell wall polysaccharides directly from single bacterial cells. Such spectra can be used to discriminate bacterial species, determine Gram type, and even assess metabolic activity through Raman–D₂O labeling (Raman-DIP). Advanced modalities such as surface-enhanced Raman scattering (SERS) and confocal Raman microspectroscopy have further improved sensitivity, allowing rapid detection of pathogens in clinical samples and phenotypic antimicrobial susceptibility testing [22–26]. Timely pathogen detection can improve infection management and reduce healthcare costs. Given the urgent need for alternative strategies to combat *K. pneumoniae* infections [21], single-cell Raman spectroscopy offers significant advantages. It allows for the identification of cellular changes at a single-cell level, aiding in understanding disease pathogenesis.

In this study, we developed a mouse model of acute pneumonia induced by *K. pneumoniae* and employed single-cell Raman spectroscopy, multicolor immunofluorescence, and exosome metabolomics. These techniques enabled the rapid identification of *K. pneumoniae* in infected tissues and provided insights into the infection mechanism. Our findings demonstrate that *K. pneumoniae* can invade airway epithelial cells early in the infection, with exosomes playing key roles in macrophage polarization and neutrophil infiltration. These results enhance our understanding of early-stage *K. pneumoniae*-induced lung infections and expand the application of Raman spectroscopy in bacterial detection and cellular analysis.

## Materials and methods

### Cell culture

THP-1 cells were sourced from FuHeng BioLogy (Shanghai, China) and cultured in RPMI 1640 medium (Gibco, NY, U.S.A.) with 10% fetal bovine serum (FBS) (BI, Israel) and 1% penicillin–streptomycin (Solarbio, Beijing). BEAS-2B and HUVEC cells were obtained from Shanghai Enzyme-linked Biotechnology Co., Ltd. (Shanghai, China) and maintained in Dulbecco’s Modified Eagle Medium (Gibco, NY, U.S.A.) with 10% FBS and 1% penicillin–streptomycin. All cells were incubated at 37°C with 5% CO_2_ in a humidified incubator (Thermo Fisher Scientific, MA, U.S.A.).

### Patient tissues

The tissues used were discarded during surgeries for lung lesions. Tissues without *K. pneumoniae* infection served as the control group, while those with the infection were used as the experimental group.

### Bacterial culture

*K. pneumoniae* subsp. pneumoniae (ATCC 13883) was sourced from Testobio Biotechnology Co., Ltd (Ningbo, China). It was cultured overnight in lysogeny broth (LB) at 37°C with shaking at 220 rpm. The cells were collected by centrifuging at 12,000 rpm for 5 min, then resuspended in phosphate-buffered saline (PBS) from Solarbio (Beijing, China). After a second centrifugation and washing, the cells were resuspended in PBS. For the infection experiments, PBS alone served as the control. The bacterial concentration was determined by measuring OD600 and adjusted to the desired colony-forming unit (CFU) concentration for each experiment.

### Fluorescent markers of *K. pneumoniae*

*K. pneumoniae* was grown in LB medium at 37°C until the OD600 reached 0.6. The culture was then diluted to an OD600 of 0.3 with fresh medium containing 0.1 mM FITC-d-Lys (Qiyue Biological Technology, Xi'an, China). Cultivation continued at 37°C until the OD600 reached 1.5. The culture was centrifuged at 12,000 rpm for 5 min to collect the bacterial pellet, which was resuspended and centrifuged again in PBS. After washing to remove excess dye, the bacteria were resuspended in endotoxin-free PBS. The suspension was placed on a slide and examined under a fluorescence microscope to confirm successful staining. These stained bacteria were then used in further experiments.

### *K. pneumoniae* infection of BEAS-2B cells

BEAS-2B cells (1 × 10^5^) were seeded in a T25 flask. Once the cells adhered, 1 × 10^7^ CFU of *K. pneumoniae* was added and incubated for 1 h to initiate infection. The medium was removed, and the cells were washed three times with PBS. A serum-free medium containing 50 μg/ml gentamicin (Solarbio, Beijing, China) was then added, and the culture was maintained for 48 h. The culture supernatant was collected afterward.

### Exosome enrichment

Prior to exosome isolation, the collected culture supernatant was subjected to sterility testing to ensure the absence of viable *K. pneumoniae*. Specifically, 100 μl of the supernatant was plated on LB agar and incubated for 24 h at 37°C, confirming no bacterial growth.

The sterile supernatant was centrifuged sequentially at 300 ***g*** for 10 min, 2000 ***g*** for 10 min, and 10,000 ***g*** for 10 min at 4°C to remove cells and debris. It was then filtered using a 0.22 μm filter (Millipore, Massachusetts, U.S.A.) to eliminate residual bacteria and large vesicles. Next, 0.5 vol of Total Exosome Isolation Reagent (Invitrogen, Carlsbad, U.S.A.) was added, and the mixture was incubated overnight at 4°C. The following day, the supernatant was centrifuged at 10,000 ***g*** for 1 h at 4°C, and the precipitate was collected for further characterization. The isolated vesicles were characterized by multiple complementary methods. Transmission electron microscopy (TEM) was used to observe the typical cup-shaped morphology of exosomes, nanoparticle tracking analysis (NTA) determined the particle size distribution, and western blot analysis confirmed the presence of exosomal markers (CD63, TSG101) while verifying the absence of bacterial or cellular debris contamination. Collectively, these results confirmed that the isolated vesicles were high-purity, host cell–derived exosomes. The total protein concentration of exosomes was quantified using the BCA Protein Assay Kit (Solarbio, Beijing, China) to ensure equal protein loading in subsequent experiments. For experiments, exosomes were used at a final concentration of 10 μg/mL. Specifically, exosomes isolated from *K. pneumoniae*-infected or control BEAS-2B cells were incubated with HUVECs and PMA-differentiated THP-1 macrophages in serum-free medium for 48 h. After incubation, HUVECs were analyzed by immunofluorescence staining and western blotting to assess the expression of tight junction proteins (ZO-1 and occludin), while macrophages were evaluated for migration (transwell assay), morphological changes (phase-contrast microscopy), and polarization marker expression (CD86, NOS2, CD206, and CD163) by q-PCR.

### Exosome metabolomics analysis

Exosomes from three independent batches of *K. pneumoniae*-infected BEAS-2B cells and control cells were sent to Biotree Biotech Co., Ltd (Shanghai, China) for metabolomic analysis. The exosome precipitate was dissolved in 1 ml of extraction solution and transferred to a 2 ml sample tube. Then, 10 μl of 1 mg/ml L-2-chlorophenylalanine was added as an internal standard. The solution underwent ultrasound sonication three times, followed by a 1 h incubation. The lysate was centrifuged at 12,000 rpm for 15 min, and the supernatant was collected after drying and re-dissolving. Metabolites in the exosomes were analyzed using ultra-high-performance liquid chromatography tandem quadrupole time-of-flight mass spectrometry (UHPLC-QTOF-MS). The raw data were processed with Leco ChromaTOF software (V4.3X). Annotation methods were applied using the R package CAMERA, and the mass spectra and retention indices were compared with the MS2 commercial database (Biotree Biotech Co., Ltd, Shanghai, China) for metabolite annotation (J. Agric. Food Chem., 68 (2020), pp. 8925–8935).

### Differentiation of THP-1 cells into macrophages

THP-1 monocytes were seeded into T25 flasks or six-well plates at a density of 1 × 10⁶ cells/ml and induced to differentiate into macrophages using 100 ng/ml phorbol 12-myristate 13-acetate (PMA; Sigma, St. Louis, U.S.A.) for 24 h. After PMA stimulation, cells were washed three times with PBS and cultured in PMA-free RPMI-1640 medium supplemented with 10% FBS for another 24 h to allow maturation and stabilization. The adherent macrophages were gently detached using a cell scraper, resuspended in serum-free medium, and counted with a hemocytometer. The resulting macrophage suspension was then adjusted to the desired density for subsequent functional assays.

### Macrophage infiltration assay

Differentiated macrophages were seeded into the upper chambers of 8 µm pore-size Transwell inserts (Corning Life Science, NY, U.S.A.) at a density of 2 × 10⁵ cells per insert in serum-free medium. Meanwhile, *K. pneumoniae*-infected BEAS-2B cells were seeded into the lower wells of 24-well plates and cultured with exosome-free medium (BI, Israel) for 24 h to generate conditioned environments. After co-culture, the non-migrated cells on the upper surface of the membranes were removed with cotton swabs, and the migrated macrophages on the lower surface were fixed in 4% paraformaldehyde and stained with Giemsa stain (Solarbio, Beijing, China). The number of migrated cells was counted in five randomly selected microscopic fields per insert, and the mean value was used for statistical analysis.

### Co-culture experiments

For co-culture assays, *K. pneumoniae*-infected BEAS-2B epithelial cells were seeded in the upper inserts of 0.4 µm pore-size chambers (Corning Life Science, NY, U.S.A.) at a density of 2 × 10⁵ cells per insert. Differentiated THP-1-derived macrophages or HUVECs were seeded in the lower wells of 24-well plates at a density of 3 × 10⁵ cells per well in exosome-free medium (BI, Israel). The co-culture system was maintained for 24 h at 37°C with 5% CO₂ to allow paracrine communication between the two cell types without direct contact. After incubation, the lower-chamber cells (macrophages or HUVECs) were collected for RNA, protein, or functional assays (e.g., cytokine quantification, endothelial permeability measurement, and gene expression analysis). All experiments were performed in triplicate.

### RNA extraction and q-PCR

Total cellular RNA was extracted using TRIzol (Invitrogen, Carlsbad, U.S.A.), and its concentration was measured with a Nanodrop (Sigma, St Louis, U.S.A.). The RNA was then reverse transcribed into cDNA using the EasyScript® One-Step gDNA Removal and cDNA Synthesis SuperMix kit (TransGen Biotech, Beijing, China). q-PCR was performed with the Hieff® q-PCR SYBR Green Master Mix (Yeasen Biotechnology Co., Ltd., Shanghai, China). The primers used were synthesized by Sangon Biotech (Shanghai, China) Co., Ltd.

**Table:** 

Gene	Forward primer (5′-3′)	Reverse primer (5′-3′)
β-actin	ACCAACTGGGACGACATGGA	GGTCTCAAACATGATCTGGGTCAT
NOS2	TTCAGTATCACAACCTCAGCAAG	TGGACCTGCAAGTTAAAATCCC
CD163	TTTGTCAACTTGAGTCCCTTCAC	TCCCGCTACACTTGTTTTCAC
CD206	TCCGGGTGCTGTTCTCCTA	CCAGTCTGTTTTTGATGGCACT
CD86	CTGCTCATCTATACACGGTTACC	GGAAACGTCGTACAGTTCTGTG
IL-6	ACTCACCTCTTCAGAACGAATTG	CCATCTTTGGAAGGTTCAGGTTG
TNF-α	CCTCTCTCTAATCAGCCCTCTG	GAGGACCTGGGAGTAGATGAG
CXCL1	ACTGAGAGTGATTGAGAGTGGAC	TGAGGGCCTGCTTCTTTCAG
CXCL2	AGCTCTGTCTGGAGCATCCAC	CAGTTAGCCTTGCCTTTGTTCAG

### Animal experiments

Female BALB/c mice (5–6 weeks old) were obtained from Beijing Vital River Laboratory Animal Technology Co., Ltd. (Beijing, China). All animal experiments were conducted at the Basic Medical College of Jilin University. Mice were lightly anesthetized with isoflurane prior to intranasal infection with *K. pneumoniae* (1 × 10⁵ CFU per mouse in 30 μl of endotoxin-free PBS). Blood and lung tissues were collected at 0.5, 1, and 3 h postinfection. Mice were killed by cervical dislocation under deep isoflurane anesthesia. All procedures complied with institutional guidelines and were approved by the Animal Ethics and Experimental Safety Committees of the Basic Medical College of Jilin University (Approval No.: 2023–454).

Peripheral blood (approximately 100 μl) was collected from the retro-orbital sinus at each time point and transferred into EDTA-coated tubes to prevent coagulation. The proportions of neutrophils, lymphocytes, monocytes, eosinophils, and basophils were determined using an automatic hematology analyzer (Mindray BC-2800Vet, Shenzhen, China). All analyses were performed within 1 h after collection, and each sample was measured in duplicate to ensure accuracy.

### Tissue immunofluorescence

The tissue was extracted and embedded in optimal cutting temperature compound (Sakura Finetek, Nagano, Japan). Sections were fixed with 4% paraformaldehyde (Solarbio, Beijing, China) for 30 min, followed by antigen retrieval and serum blocking. After gently removing the blocking solution, the prepared antibodies were applied to the tissue and incubated overnight at 4°C. The antibodies used included anti-F4/80 (Cell Signaling Technology, 70076, 1:200), anti-CK8 (Proteintech, 17514–1-AP, 1:200), anti-iNOS (Proteintech, 18985–1-AP, 1:100), anti-CD206 (Abcam, ab64693, 1:100), anti-ZO-1 (Proteintech, 21773–1-AP, 1:200), and anti-MPO (Abcam, ab208670, 1:100). The following day, the primary antibodies were washed off, and either anti-rabbit IgG (H + L) antibody (SeraCare, 5220–0336, 1:500) or anti-mouse IgG (H + L) antibody (SeraCare, 5220–0341, 1:500) was added and incubated at room temperature for 50 min. This was followed by the application of AF488 tyramide, Cy3 tyramide, or Cy5 tyramide for 20 min at room temperature. Finally, nuclei were stained with DAPI and images were captured using a fluorescence microscope.

### Western blotting

RIPA lysate was added to exosomes and whole-cell precipitate to extract total protein. Protein concentration was measured using a BCA kit (Solarbio, Beijing, China) and adjusted to 1 mg/ml with PBS and 5 × loading buffer (Solarbio, Beijing, China). For the western blot experiments, 10 μl of each sample was used. The primary antibodies included anti-ZO-1 (Proteintech, 21773–1-AP, 1:1000), anti-occludin (Proteintech, 27260–1-AP, 1:1000), anti-NF-kB p65 (Abcam, ab207297, 1:1000), anti-NF-kB p65 (phospho S536) (Abcam, ab86299, 1:500), anti-CD63 (Proteintech, 67605–1-Ig, 1:1000), anti-TSG101 (Proteintech, 67381–1-Ig, 1:1000), and anti-GAPDH (Abcam, ab181602, 1:5000). The secondary antibodies used were anti-mouse IgG (H + L) cross-adsorbed secondary antibody (Invitrogen, G-21040, 1:5000) or anti-rabbit IgG (H + L) cross-adsorbed secondary antibody (Invitrogen, G-21234, 1:5000).

### Acquisition of Raman spectra of cells and tissues

Cell samples were fixed with 4% paraformaldehyde and washed three times with sterile water. Each wash involved centrifugation at 5000 rpm for 5 min, followed by the addition of 1 ml of sterile water to the collected precipitate. Tissue cryosections were directly placed onto the chip. The samples were then placed on a confocal Raman spectrometer (P300, Hooke Instrument) for Raman scanning. The acquisition parameters were a 532 nm excitation wavelength, 1200 grating, 5 mW power, 8 s integration time, and 0.5 μm step size, covering a spectral range of 500–2000 cm^−1^.

### Raman mapping analysis

#### Data preprocessing

Raman scan data often include interference such as spectrometer noise and fluorescence background, which can affect the Raman peaks or features of interest. Before analysis, these data need preprocessing. In this study, Hooke intP Raman analysis software was used to preprocess each single-point scan in the mapping data. This involved smoothing and filtering using the Savitzky-Golay method, with a window width of 5 and third-order polynomial fitting. The AirPLS method was employed to remove background signals, with a Lambda value set to 100 and a maximum of 15 iterations. Normalization was performed using the Min-Max method.

#### Raman mapping analysis

The results are presented as heat maps of the characteristic peak area. The linear background method total peak area (TPAs) was used to calculate the characteristic peak area of the preprocessed single-point spectral data. To minimize noise impact on the background (L, R) of the characteristic peak’s boundaries, the trapezoidal area connecting L, R, and the spectral background was recorded as the background area. The integral value between L and R was recorded as the total area. The characteristic peak area was determined by subtracting the background area from the total area. These values were then visualized as heat maps, with a blue to red gradient indicating increasing characteristic peak intensity and abundance.

### Statistics

Sample size calculations were not performed using statistical methods. All experiments were conducted with at least three independent biological replicates (*n* = 3) unless otherwise stated. Statistical analyses were conducted with GraphPad software, utilizing one-way analysis of variance with Bonferroni correction for multiple comparisons, or an unpaired two-tailed Student’s *t*-test. Error bars represent the standard deviation (SD) of the means. Statistical significance is denoted as follows: ns, not significant (*P*≥0.05); *P<0.05; **P<0.01; ***P<0.001.

## Results

### Early invasion of airway epithelial cells by *K. pneumoniae* in acute pneumonia

**Figure 1:**
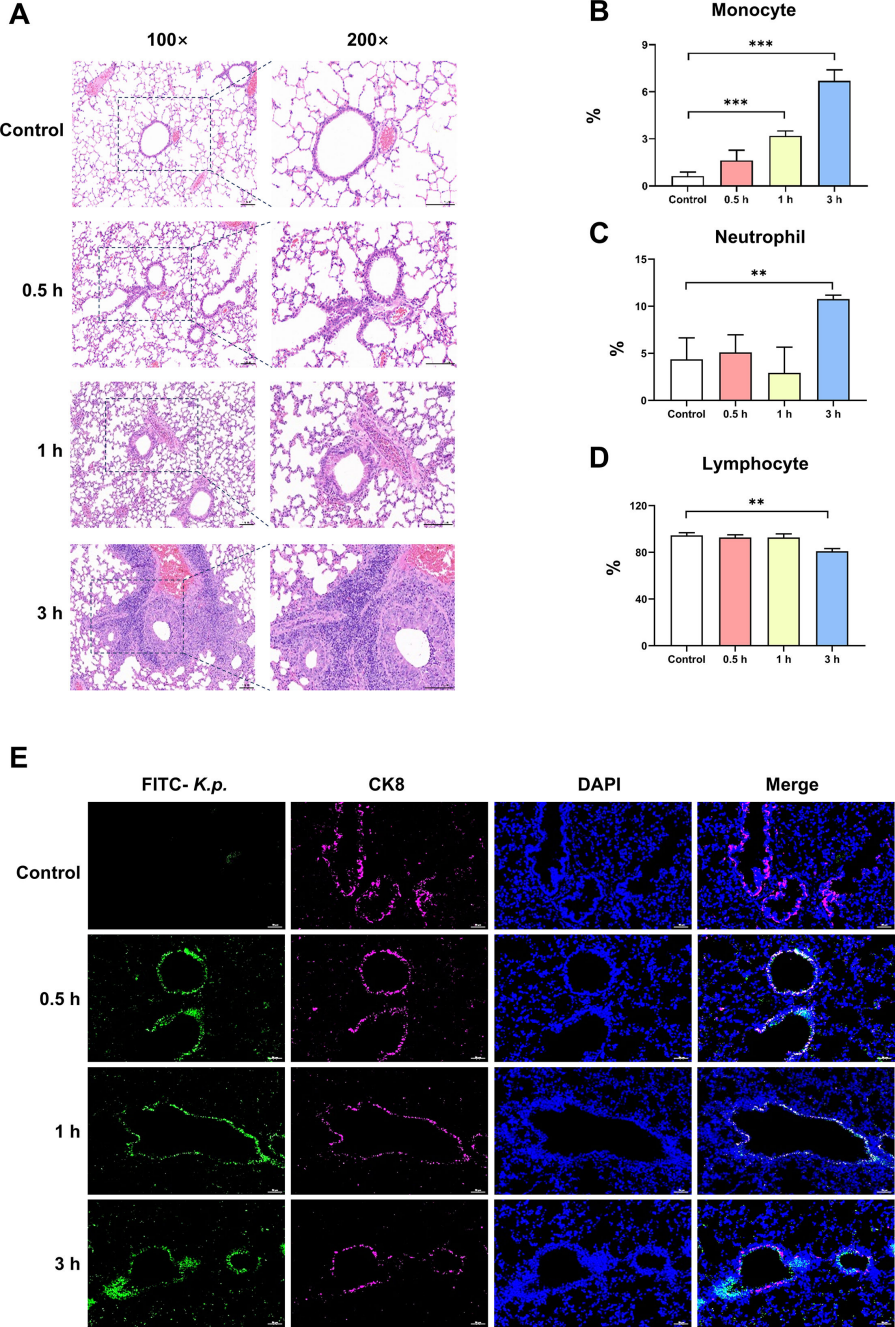
Cellular and tissue response to FITC-conjugated *K. pneumoniae* infection. (**A**) Histological analysis of lung tissue at different time points (Control, 0.5 h, 1 h, 3 h) postinfection, showing tissue infiltration at 100× and 200× magnification. Scale bar: 100 μm. (**B-D**) Quantitative analysis of immune cell populations in the lung tissue at various time points postinfection: (**B**) Monocytes, (**C**) Neutrophils, (**D**) Lymphocytes. Statistical significance is indicated: **P<0.01, ***P<0.001. Data represent mean ± SD from three independent experiments (*N* = 3). (**E**) Immunofluorescence images showing co-localization of FITC-conjugated *K. pneumoniae* (green), CK8 (magenta), and nuclei (DAPI, blue) in lung tissue at different time points (Control, 0.5 h, 1 h, 3 h). Merged images illustrate the spatial distribution. Scale bar: 100 μm.

In the early stages of *K. pneumoniae*-induced acute pneumonia, the bacteria initially invade airway epithelial cells. To investigate the mechanism by which *K. pneumoniae* causes pneumonia, we labeled the bacteria with FITC-d-Lys during culture. These labeled bacteria were then introduced into the nasal cavity of mice to establish a model of acute pulmonary infection (Supplementary Figure S1A). Then, fluorescence microscopy confirmed that most labeled *K. pneumoniae* exhibited green fluorescence (Supplementary Figure S1B). Hematoxylin and eosin (H&E) staining revealed significant inflammatory cell infiltration and airway wall thickening as early as 0.5 h postinfection, compared with control mice (Figure 1A). By 3 h, there was a marked increase in the number of inflammatory cells and further airway thickening. We also measured blood levels of various immune cells. The proportion of monocytes began to rise at 0.5 h postinfection (Figure 1B), with a significant increase in neutrophils observed at 3 h (Figure 1C). Lymphocyte levels showed a slight increase over time (Figure 1D), while eosinophil (Supplementary Figure S1C) and basophil (Supplementary Figure S1D) levels remained relatively stable. Microscopic observations of frozen lung sections showed green fluorescence in the shape of the airway, indicating bacterial presence. To confirm *K. pneumoniae* entry into airway epithelial cells, CK8 immunofluorescence staining was used. The results showed co-localization of FITC-labeled *K. pneumoniae* with CK8, particularly at 0.5 h postinfection, indicating bacterial entry into epithelial cells (Figure 1E). While the overlap decreased slightly over time, strong co-localization persisted (Supplementary Figure S1E). *In vitro* experiments with BEAS-2B cells, a human airway epithelial cell line, further confirmed that *K. pneumoniae* could enter these cells (Supplementary Figure S1F). These findings validate the acute pneumonia model and demonstrate that *K. pneumoniae* initiates infection by entering epithelial cells.

### Disruption of vascular endothelial barrier and M1 macrophage polarization induced by *K. pneumoniae* infection

**Figure 2:**
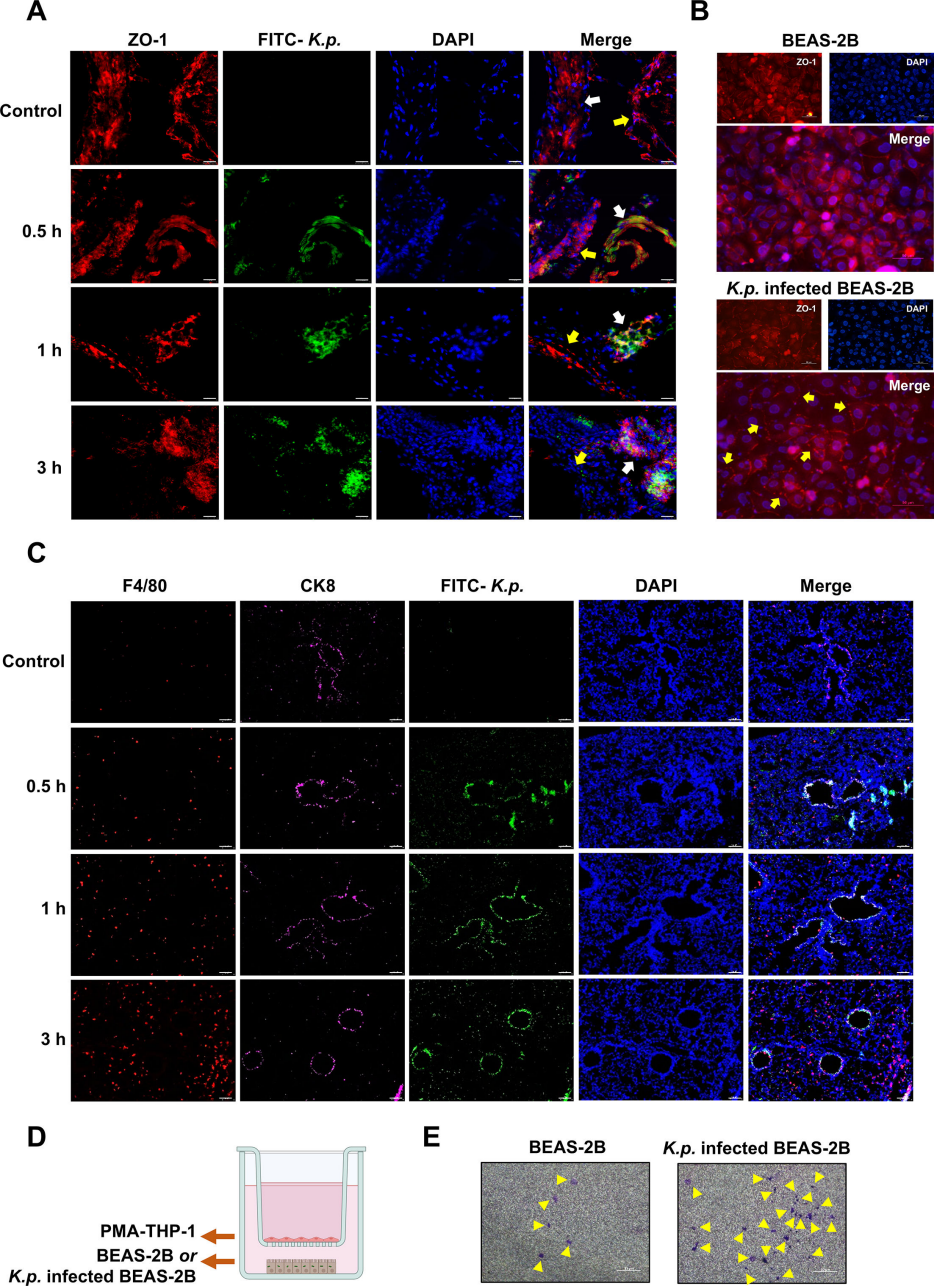
Junctional and immune response to *K. pneumoniae* infection in lung tissue and BEAS-2B cells. (**A**) Immunofluorescence images showing ZO-1 (red), FITC-conjugated *K. pneumoniae* (green), and nuclei (DAPI, blue) in lung tissue at different time points (Control, 0.5 h, 1 h, 3 h). Merged images highlight cell junction integrity. Scale bar: 25 μm. (**B**) Representative immunofluorescence images of BEAS-2B cells and HUVEC cells co-cultured with *K. pneumoniae*-infected BEAS-2B cells, showing ZO-1 (red) and nuclei (DAPI, blue) staining. Arrows indicate disrupted or discontinuous junctional ZO-1 localization, reflecting compromised tight-junction integrity after infection. Scale bar: 50 μm. (**C**) Immunofluorescence images showing F4/80 (red), CK8 (magenta), FITC-conjugated *K. pneumoniae* (green), and nuclei (DAPI, blue) in lung tissue at various time points. Merged images illustrate immune cell infiltration. Scale bar: 100 μm. (**D**) Schematic diagram of the co-culture setup used for the experiments with PMA-THP-1, BEAS-2B, or K.p. infected BEAS-2B cells. (**E**) Bright field images of BEAS-2B and K.p. infected BEAS-2B cells, highlighting morphological differences. Scale bar: 50 μm.

**Figure 3:**
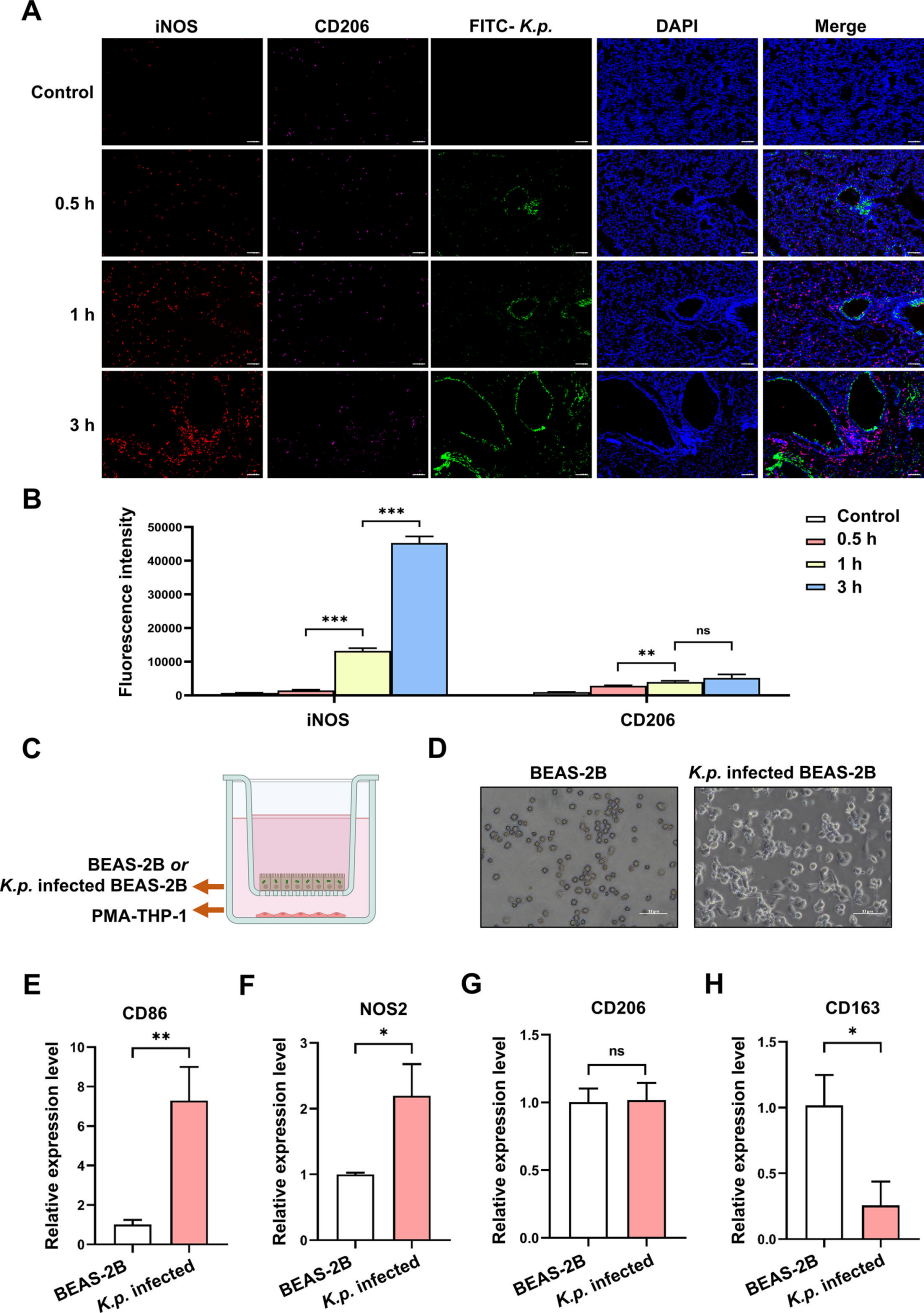
Macrophage polarization and response to *K. pneumoniae* infection. (**A**) Immunofluorescence images showing expression of iNOS (red) and CD206 (green) in lung tissue at different time points (Control, 0.5 h, 1 h, 3 h) postinfection with FITC-conjugated *K. pneumoniae*. Nuclei are stained with DAPI (blue). Scale bar: 100 μm. (**B**) Quantitative analysis of fluorescence intensity for iNOS and CD206 at various time points. Statistical significance is indicated: **P<0.01, ***P<0.001. Data represent mean ± SD from three biological replicates (*n* = 3). (**C**) Schematic of the co-culture setup with BEAS-2B or K.p. infected BEAS-2B cells and PMA-THP-1 macrophages. (**D**) Phase contrast images of BEAS-2B and K.p. infected BEAS-2B cells, showing morphological changes. Scale bar: 50 μm. (**E-H**) Relative mRNA expression levels of macrophage polarization markers: (**E**) CD86 and (**F**) NOS2 (M1 markers); (**G**) CD206 and (**H**) CD163 (M2 markers) in macrophages co-cultured with exosomes derived from BEAS-2B cells or *K. pneumoniae*-infected BEAS-2B cells. Statistical significance is indicated: *P*<0.05, *P < 0.01, **P < 0.001. All data are representative of three independent experiments (*n* = 3).

Further examination of lung tissue changes induced by *K. pneumoniae* entry into airway epithelial cells revealed significant disruptions. Immunofluorescence staining showed a marked decrease in the expression of the tight junction protein ZO-1 in vascular endothelial cells surrounding the airway, indicating a gradual breakdown of the vascular endothelial barrier as infection progressed (Figure 2A). This was corroborated by in vitro experiments, where co-culturing *K. pneumoniae*-infected BEAS-2B cells with HUVEC cells led to reduced ZO-1 expression and compromised tight junctions (Figure 2B). Given the increase in blood monocytes and the compromised vascular barrier following *K. pneumoniae* infection, we investigated macrophage infiltration at the infection site. Immunofluorescence staining showed increased macrophage infiltration around the airway, which intensified over time (Figure 2C). *In vitro*, PMA-treated THP-1 cells co-cultured with *K. pneumoniae*-infected BEAS-2B cells (Figure 2D) exhibited enhanced infiltration ability, as demonstrated by a transwell assay (Figure 2E). Macrophages were categorized into M1 and M2 types based on iNOS and CD206 expression. Immunofluorescence results indicated a progressive increase in iNOS expression around the infected airway, with no significant change in CD206 expression (Figure 3A). Quantitative fluorescence analysis confirmed a significant rise in iNOS expression (Figure 3B), indicating a predominance of M1 macrophages. *In vitro* studies further supported these findings. PMA-treated THP-1 cells co-cultured with *K. pneumoniae*-infected BEAS-2B cells (Figure 3C) showed altered morphology (Figure 3D). qPCR results revealed increased expression of CD86 and NOS2, markers of M1 polarization, while CD206 and CD163 levels showed no significant change or decreased (Figure 3E–H). These results suggest that macrophages are polarized towards the M1 phenotype during *K. pneumoniae* infection.

### Raman spectroscopic analysis of airway epithelial changes following *K. pneumoniae* infection

**Figure 4:**
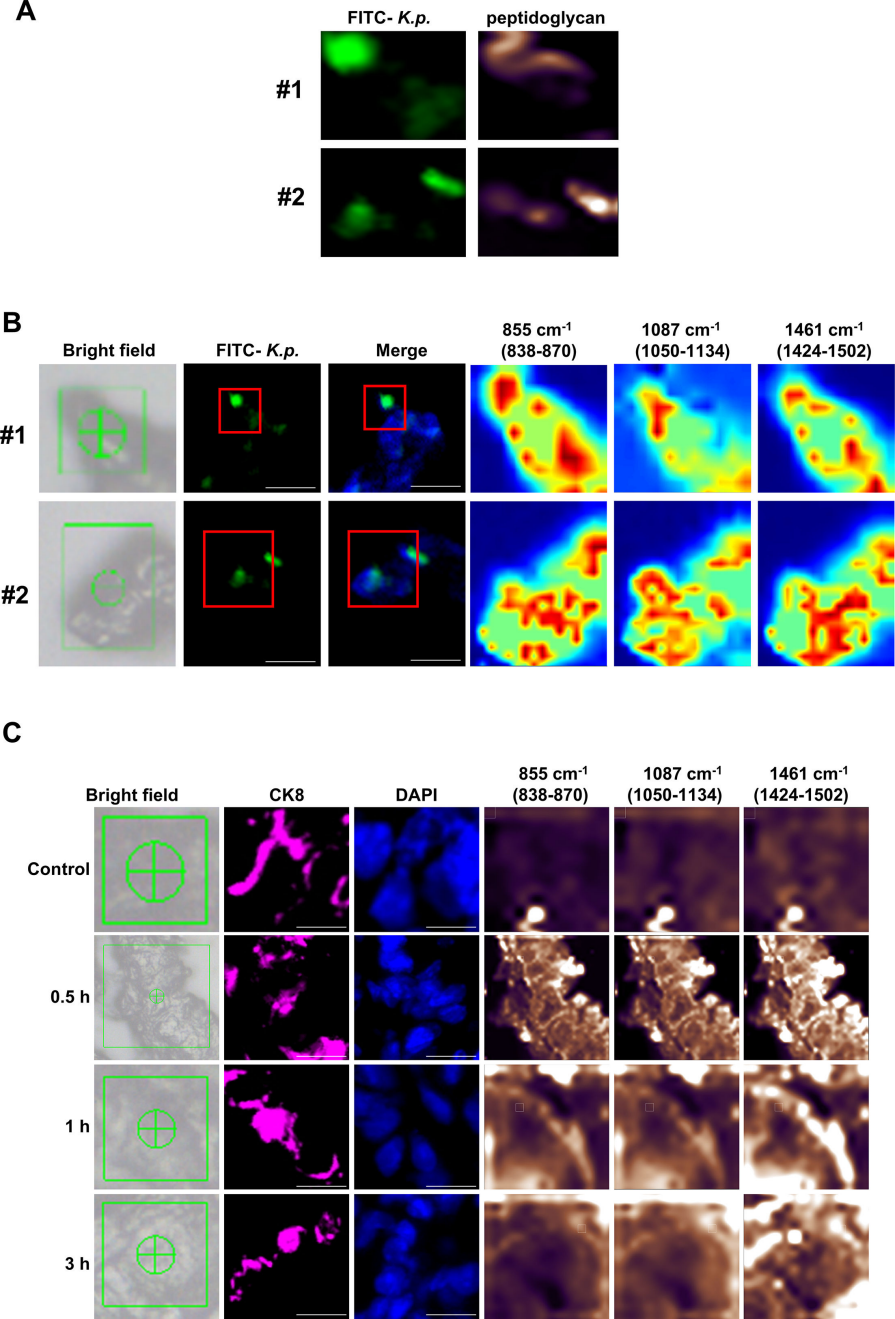
Raman spectroscopy analysis of *K. pneumoniae* and peptidoglycan. (**A**) Raman imaging showing FITC-conjugated *K. pneumoniae* and peptidoglycan in two different samples (#1 and #2). (**B**) Bright field and Raman spectral images of two samples (#1 and #2), showing distribution of FITC-K.p. and corresponding Raman shifts. Scale bar: 10 μm. (**C**)Time-course analysis of Raman spectral changes in lung tissue at different time points (Control, 0.5 h, 1 h, 3 h) postinfection, with corresponding immunofluorescence images showing CK8 and DAPI staining. Scale bar: 10 μm.

To explore how infection by *K. pneumoniae* affects airway epithelial cells, we used Raman spectroscopy to examine the resulting damage to the vascular endothelial barrier and macrophage dynamics. The technique successfully identified bacterial peptidoglycan through characteristic spectral peaks, with heat maps confirming the bacteria’s location as seen with FITC labeling (Figure 4A). Further spectral analysis at infection sites showed increased peak areas at 838–870 cm⁻¹, 1050–1134 cm⁻¹, and 1424–1502 cm⁻¹. These peaks correspond to 1,4-glycosidic bonds in polysaccharides and C-C and CH₂ bonds in lipids, indicating their elevated expression at infection sites (Supplementary Figure S2A and B). Heat maps corroborated these findings, showing increased polysaccharide and lipid expression in infected cells (Figure 4B). Comparisons with control tissue revealed that polysaccharide and lipid expressions were consistently higher in infected tissues, and this increase persisted at 0.5, 1, and 3 h post-infection in airway epithelial cells (Figure 4C, S2C). These observations indicate a significant up-regulation of these substances following *K. pneumoniae* infection.

### Role of exosomes in *K. pneumoniae*-induced cellular changes

Single-cell Raman spectroscopy demonstrated increased polysaccharide and lipid levels in *K. pneumoniae*-infected epithelial cells, alongside damage to the vascular endothelial barrier, macrophage infiltration, and M1-like polarization. Given the importance of exosomes in intercellular communication, we investigated their role in this process. Exosomes from BEAS-2B cells and those infected with *K. pneumoniae* were isolated and analyzed using transmission electron microscopy, nanoparticle tracking analysis, and Western blot (Supplementary Figure S3A, S3B, S3C), confirming intact morphology and high concentration. Immunofluorescence staining with PKH67 indicated that both types of exosomes were absorbed by PMA-treated THP-1 cells and played a role in regulation (Supplementary Figure S3D).

**Figure 5:**
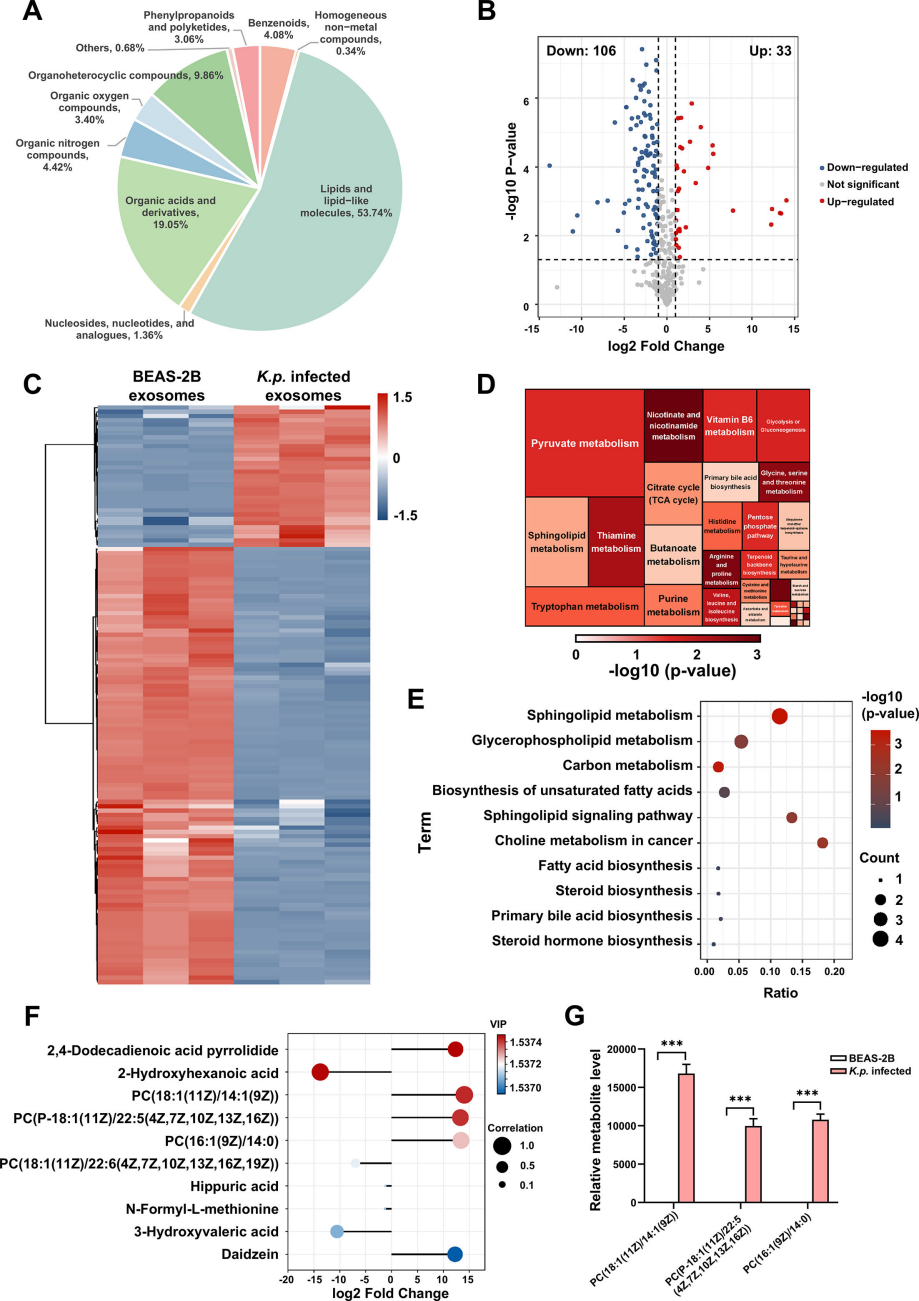
Metabolomic profiling of exosomes from *K. pneumoniae*-infected epithelial cells. (**A**) Metabolite composition of exosomes, highlighting lipids and lipid-like molecules as the most abundant category, followed by organic acids and derivatives. (**B**) Volcano plot showing significant up-regulation and down-regulation of metabolites in *K. pneumoniae*-infected exosomes compared with controls. Results are based on three independent biological replicates (*n* = 3). (**C**) Heatmap indicating differential expression of metabolites between BEAS-2B exosomes and those from *K. pneumoniae*-infected cells. (**D**) Enrichment analysis of metabolic pathways, with significant findings in sphingolipid and pyruvate metabolism. (**E**) Pathway analysis demonstrating significant enrichment in lipid-related metabolic pathways, including sphingolipid and glycerophospholipid metabolism. (**F**) VIP plot of the top 10 differential metabolites, highlighting those contributing to differences between groups. (**G**) Bar graph showing significantly increased levels of specific phosphatidylcholines in *K. pneumoniae*-infected exosomes compared with controls, indicating enhanced lipid metabolism. Data are expressed as mean ± SD from three independent replicates (*n* = 3).

Exosomes extracted from infected and control epithelial cells were analyzed with UHPLC-OE-MS, identifying 366 metabolites, including lipids, nucleotides, organic acids, and more. Lipids and lipid-like molecules (53.74%) and organic acids and derivatives (19.05%) were the most abundant. Organoheterocyclic compounds accounted for 9.86% (Figure 5A). Further analysis revealed significant differences between the two groups, with 106 metabolites down-regulated and 33 up-regulated postinfection (Figure 5B). Differential metabolites were identified using the VIP from an OPLS-DA model, revealing differences between *K. pneumoniae*-infected and control exosomes (Figure 5C). Significant enrichment was noted in pyruvate and sphingomyelin metabolism (Figure 5D). Lipid-related pathways such as sphingolipid and glycerophospholipid metabolism were notably enriched (Figure 5E). The top 10 differential metabolites highlighted included three phosphatidylcholines with significant expression increases (Figure 5F and G). *K. pneumoniae* infection also prompted vascular barrier destruction and macrophage changes.

### Impact of *K. pneumoniae*-infected exosomes on endothelial barrier integrity and macrophage polarization

**Figure 6:**
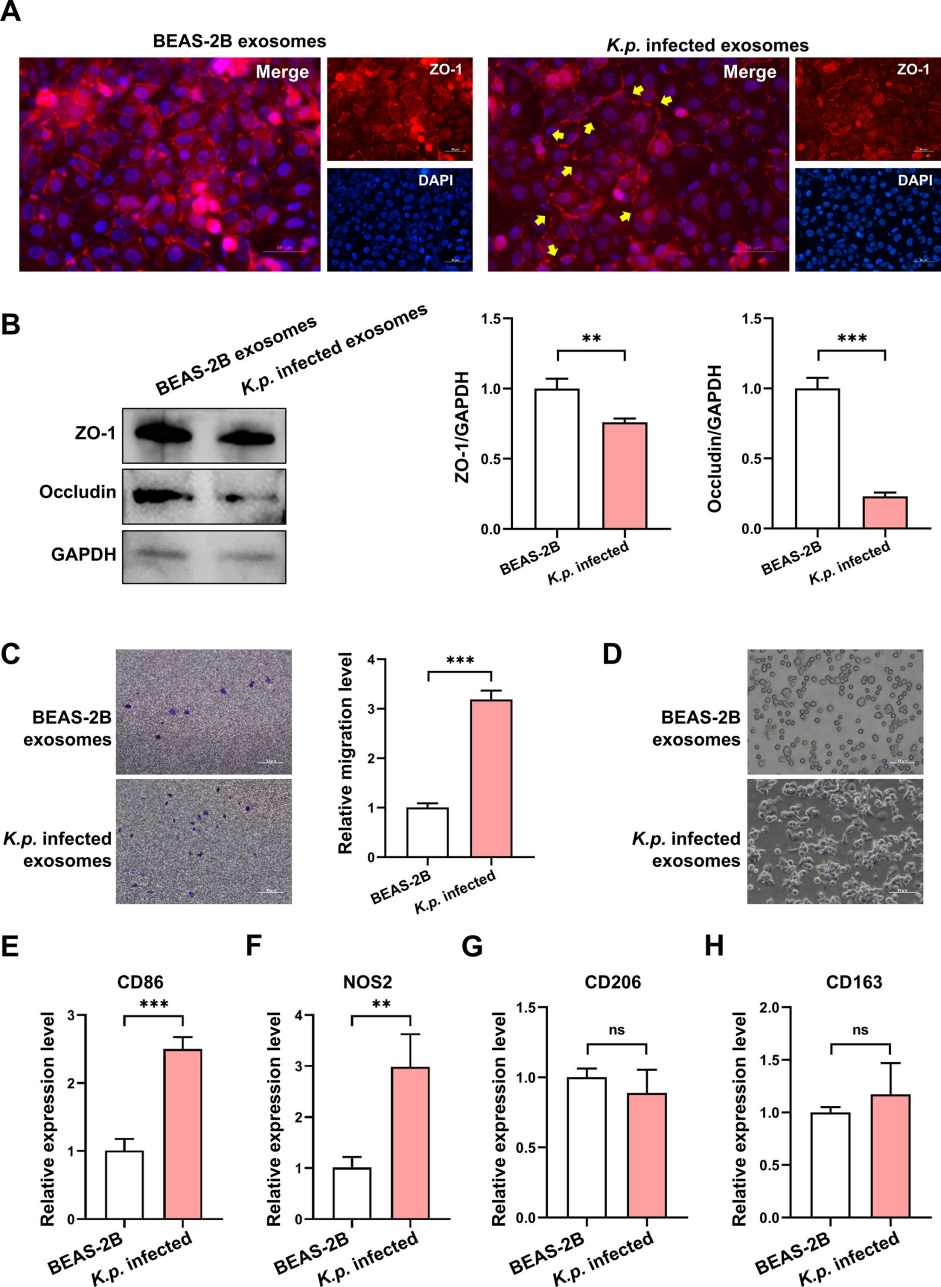
Effects of *K. pneumoniae*-infected exosomes on endothelial barrier and macrophage polarization. (**A**) Immunofluorescence images showing the reduction of ZO-1 expression in HUVEC cells after treatment with *K. pneumoniae*-infected exosomes, indicating disrupted tight junctions. Scale bar: 50 μm. (**B**) Western blot analysis quantifying the decreased expression of ZO-1 and occludin in HUVEC cells incubated with *K. pneumoniae*-infected exosomes compared with controls. **P<0.01, ***P<0.001. Each experiment was performed independently three times (*n* = 3). (**C**) Transwell assay demonstrating enhanced macrophage infiltration when co-cultured with *K. pneumoniae*-infected exosomes. Scale bar: 50 μm. (**D**) Morphological changes in macrophages incubated with *K. pneumoniae*-infected exosomes, showing increased elongation compared with controls. Scale bar: 50 μm. (**E-H**) qPCR analysis of macrophage polarization markers. Increased expression of CD86 and NOS2 indicates polarization towards M1 macrophages, while CD206 and CD163 levels remain unchanged. **P<0.01, ****P<0.0001, ns: not significant. All quantitative data are from three independent experiments (*n* = 3).

To investigate how exosomes from *K. pneumoniae*-infected airway epithelial cells affect the vascular endothelial barrier, these exosomes and controls were incubated with vascular endothelial cells. Immunofluorescence analysis showed that *K. pneumoniae*-infected exosomes significantly reduced the expression of the tight junction protein ZO-1, disrupting the connections between HUVEC cells (Figure 6A). Further examination confirmed decreased levels of ZO-1 and occludin, indicating barrier compromise (Figure 6B). We also assessed the role of these exosomes in macrophage infiltration and polarization. Using a transwell assay, we found that *K. pneumoniae*-infected exosomes enhanced macrophage infiltration (Figure 6C). Morphological analysis demonstrated that macrophages exposed to these exosomes showed greater extension (Figure 6D). q-PCR analysis revealed increased expression of CD86 and NOS2, markers of M1 macrophage polarization, while CD206 and CD163 did not show significant changes (Figure 6E–H). These findings suggest that exosomes from *K. pneumoniae*-infected cells facilitate vascular barrier disruption and promote macrophage infiltration and M1 polarization.

### Activation of NF-κB pathway in macrophages by *K. pneumoniae*-infected exosomes

**Figure 7:**
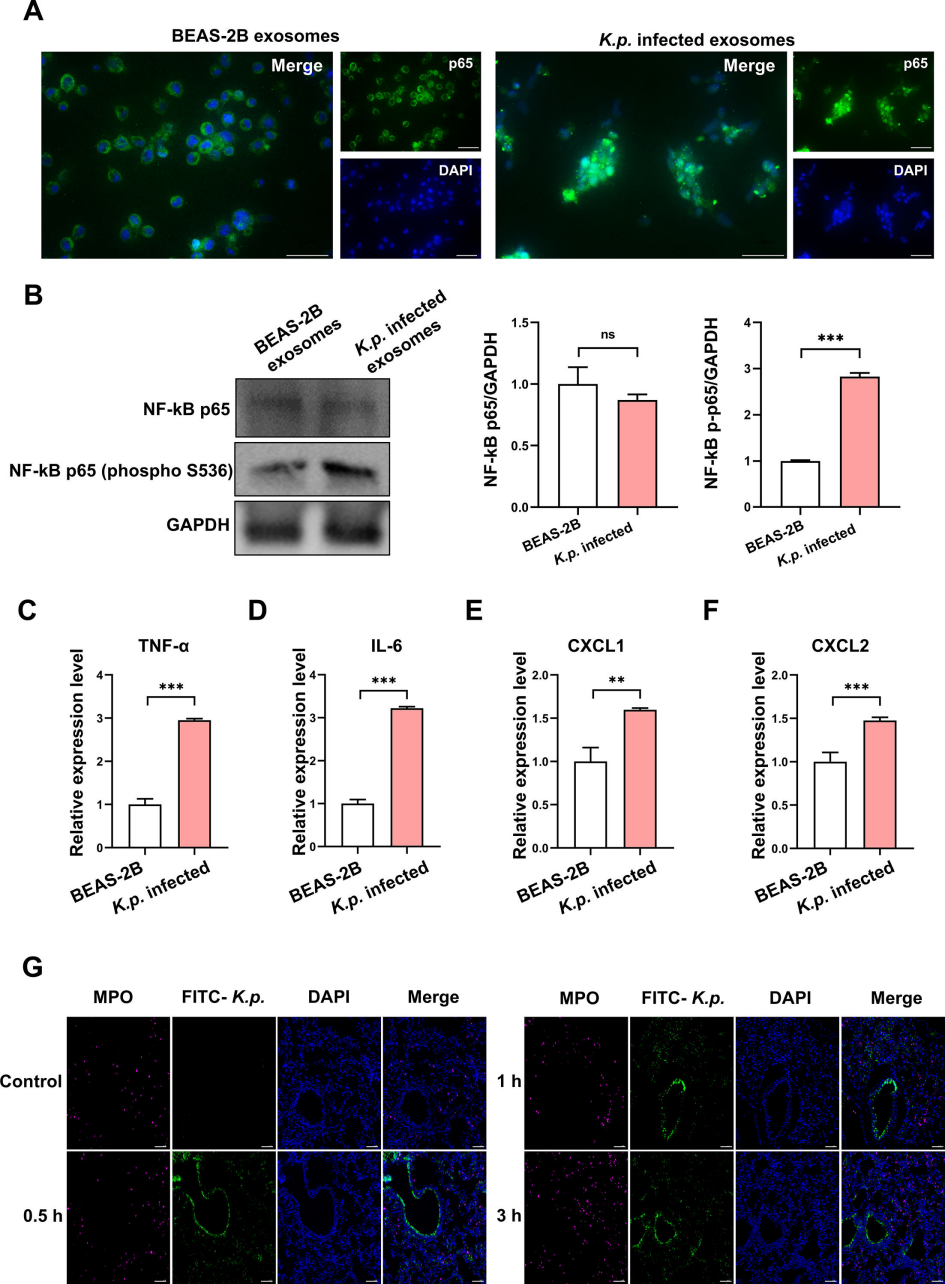
Activation of the NF-κB pathway and inflammatory response by exosomes from *K. pneumoniae*-infected cells. (**A**) Immunofluorescence images showing nuclear translocation of p65 in macrophages treated with *K. pneumoniae*-infected exosomes, indicating NF-κB activation. Scale bar: 50 μm. (**B**) Western blot analysis demonstrating increased phosphorylation of NF-κB p65 in macrophages stimulated with *K. pneumoniae*-infected exosomes compared with controls. **P<0.01, ***P<0.001. (**C-F**) qPCR results showing elevated expression levels of inflammatory cytokines TNF-α, IL-6, and chemokines CXCL1 and CXCL2 in macrophages exposed to *K. pneumoniae*-infected exosomes. **P<0.01, ***P<0.001. Data are expressed as mean ± SD from three independent replicates (*n* = 3). (**G**) Immunofluorescence staining indicating an increase in MPO expression and neutrophil infiltration at 3 h postinfection, highlighting the inflammatory response induced by *K. pneumoniae* infection. Scale bar: 100 μm.

Cauvi et al. discovered that phosphatidylcholine liposomes can drive macrophages towards an inflammatory state by activating the NF-κB pathway, increasing cytokines and chemokines, and rapidly attracting neutrophils [27]. Similarly, our study found an increase in neutrophils in the blood 3 h post-*K. pneumoniae* infection, coupled with elevated phosphatidylcholine levels in epithelial cell exosomes. This prompted us to explore whether the exosomal entry into macrophages triggers NF-κB activation. We observed an increase in cytokines and chemokines, along with rapid neutrophil recruitment. Immunofluorescence revealed nuclear translocation of P65 in macrophages stimulated by *K. pneumoniae*-infected exosomes (Figure 7A). Additionally, western blot analyses showed enhanced phosphorylation of NF-κB p65 (Figure 7B), suggesting pathway activation. We further analyzed cytokine and chemokine levels: TNF-α (Figure 7C), IL-6 (Figure 7D), CXCL1 (Figure 7E), and CXCL2 (Figure 7F) were elevated in macrophages exposed to these exosomes. Tissue immunofluorescence indicated that although MPO levels did not change significantly at 0.5 and 1 h, they increased alongside neutrophil infiltration by 3 h postinfection (Figure 7G). This indicates that exosomes from *K. pneumoniae*-infected epithelial cells can enter macrophages and activate the NF-κB signaling pathway, boosting TNF-α, IL-6, CXCL1, and CXCL2 levels, leading to neutrophil infiltration and enhancing inflammation.

### Analysis of tissue changes and lipid expression in *K. pneumoniae*-infected lungs

**Figure 8:**
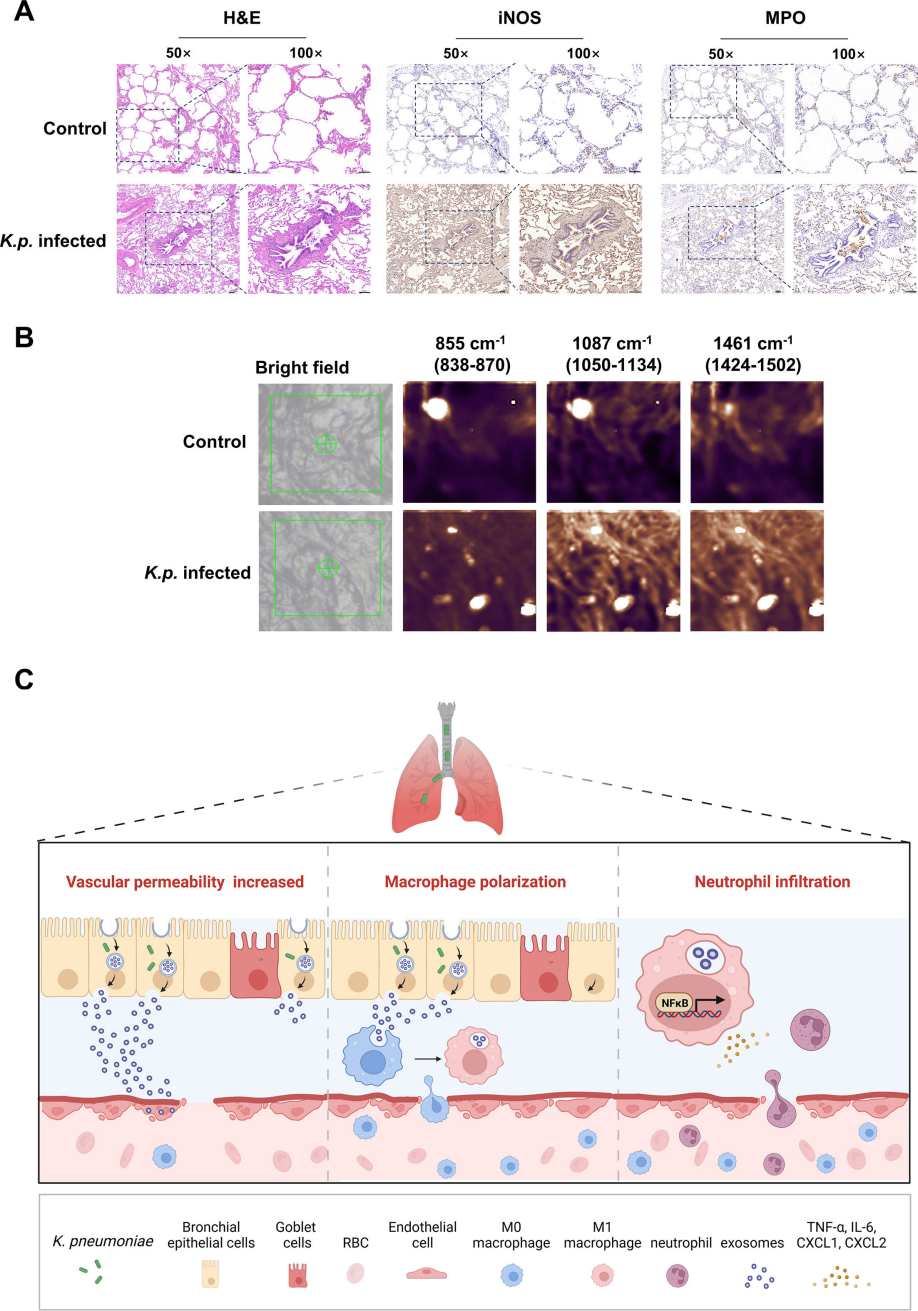
Impact of *K. pneumoniae* infection on lung tissue and immune response. (**A**) H&E staining and immunohistochemical analysis of lung tissues from control (*n* = 2) and *K. pneumoniae*-infected patients (*n* = 2). Infected tissues exhibit disordered alveolar structures, thickened airway walls, and increased iNOS and MPO expression, indicating enhanced inflammation. Scale bar: 100 μm. (**B**) Single-cell Raman spectroscopy of control and infected lung tissues showing heightened lipid expression at specific peaks (855 cm⁻¹, 1087 cm⁻¹, 1461 cm⁻¹) postinfection, suggesting changes in lipid metabolism associated with infection. (**C**) Schematic representation of *K. pneumoniae*-induced changes in the lung: increased vascular permeability, macrophage polarization to the M1 phenotype, and neutrophil infiltration leading to an amplified inflammatory response.

H&E staining of *K. pneumoniae*-infected tissues compared with controls showed disordered alveolar structures, thickened airway walls, and increased inflammatory infiltration around the airways (Figure 8A). Immunohistochemistry indicated elevated levels of iNOS and MPO, alongside increased macrophage infiltration and M1 polarization. Furthermore, single-cell Raman spectroscopy of patient tissues revealed heightened expression of lipid peaks postinfection (Figure 8B). These findings confirm the validity of our detection method in patient tissues, indicating that increased lipid expression in epithelial cells may amplify the inflammatory response.

## Discussion

Our study revealed that exosomes released by *K. pneumoniae*-infected airway epithelial cells are significantly enriched in phosphatidylcholine. These exosomes disrupt the vascular endothelial barrier, recruit macrophages, and promote their polarization to M1-type macrophages. They also activate the NF-κB signaling pathway and increase chemokine expression in macrophages that ingest the exosomes, leading to neutrophil infiltration. This confirms the critical role of exosomes in *K. pneumoniae* infection.

Bacterial resistance is a growing global challenge. Rational antibiotic use is essential to maintain their effectiveness [28]. Rapid pathogen identification is crucial to minimize patient risk and provide targeted antibiotics, reducing irrational antibiotic use [29]. Traditional detection methods like bacterial culture, PCR, and ELISA each have drawbacks. Bacterial culture is time-consuming and prone to contamination [30], PCR is susceptible to sample contamination and is relatively expensive, and immunoassays require many clinical samples and are less sensitive [31].

In current clinical practice, the ‘gold standard’ for bacterial identification remains phenotypic and biochemical identification combined with antimicrobial susceptibility testing (AST) or MALDI-TOF analysis after culturing pure isolates, which typically requires 24–72 h. While molecular approaches such as PCR, 16S rRNA sequencing, or whole-genome sequencing (WGS) can improve specificity, they are target-dependent, costly, and labor-intensive, which limits their routine clinical applicability [32]. In contrast, Raman spectroscopy enables label-free, culture-independent, and phenotypic bacterial detection within minutes to hours. When coupled with Raman–D₂O labeling (Raman-DIP), it can perform rapid phenotypic antimicrobial susceptibility testing by assessing bacterial metabolic activity via the C–D signature.

A single Raman spectrum encodes multiple molecular features—nucleic acids, proteins, lipids, and polysaccharides—allowing simultaneous inference of bacterial species, Gram status, and metabolic state. Furthermore, techniques such as surface-enhanced Raman scattering (SERS) and resonance Raman spectroscopy can enhance sensitivity and reduce acquisition time. At the single-cell level, Raman spectroscopy can detect intrapopulation heterogeneity and identify tolerant or persister bacterial subpopulations that are often missed by bulk assays. Importantly, once the instrument is in place, the per-test cost is very low, and the system is compatible with portable or microfluidic-based platforms, offering a practical and cost-effective diagnostic option for rapid triage in clinical settings.

Optical techniques like Raman spectroscopy and single-particle imaging are promising due to their high sensitivity, simplicity, and low cost for label-free bacterial detection. In our study, single-cell Raman spectroscopy enabled rapid bacterial detection in tissues and cells, providing a better approach for detecting bacteria in clinical samples like epithelial cells from puncture tissues or throat swabs.

Our findings align with previous studies highlighting the role of exosomes in pulmonary infections. For instance, in sepsis-associated acute lung injury (ALI), exosomal miR-30d-5p from neutrophils induces the polarization of M1 macrophages and triggers macrophage pyroptosis [33]. Similarly, exosomes rich in miR-155 were found to increase M1 macrophages and cause lung inflammation in mice [34]. These studies support our findings that exosomes play a significant role in immune response modulation during bacterial infections.

One limitation of our study is the use of a model system, which may not fully replicate human responses. Future research should focus on validating these findings in human tissues and clinical settings. Additionally, exploring the specific molecular mechanisms by which exosomes influence macrophage behavior could provide deeper insights.

Raman spectroscopy holds potential for improving rapid bacterial detection and aiding personalized medicine. Its application could extend beyond infection detection to identifying bacteria within tumors, which could revolutionize cancer treatment and drug screening. For example, bacteria within tumor cells play important roles in tumor metastasis and colonization [35]. Detecting these bacteria could lead to more targeted therapies.

We anticipate that our findings will aid in developing new diagnostic tools and therapeutic strategies. Implementing Raman spectroscopy in clinical settings could face challenges such as ensuring accuracy and integrating with existing diagnostic workflows. However, its potential for rapid, label-free detection makes it a promising tool for enhancing clinical diagnostics.

## Conclusion

In conclusion, our study highlights the significant role of exosomes in *K. pneumoniae* infection and demonstrates the potential of single-cell Raman spectroscopy for rapid bacterial detection. These findings contribute to the development of new diagnostic and therapeutic strategies, addressing the growing challenge of bacterial resistance and improving patient outcomes.

**Clinical perspectives**

- *Klebsiella pneumoniae* is a major pathogen responsible for severe pulmonary infections, but its early infection mechanisms remain poorly understood.
- The study revealed that exosomes derived from infected epithelial cells contain elevated levels of phosphatidylcholine, which disrupt endothelial tight junctions, promote M1 macrophage polarization, and activate the NF-κB pathway, thereby enhancing inflammation and neutrophil recruitment.
- These findings uncover a novel exosome-mediated mechanism contributing to the early stages of *K. pneumoniae* infection and highlight potential therapeutic targets for controlling disease progression.

## Supplementary material

online supplementary material 1.

## Data Availability

All data supporting the findings of this study are provided within the article and its Supplementary Information. The exosome metabolomics data have been deposited in the Open Archive for Miscellaneous Data (OMIX) under accession number OMIX005255 [36]. The corresponding dataset is available at: https://ngdc.cncb.ac.cn/omix/release/OMIX005255 [36]. Additional datasets generated and/or analyzed during the current study are available from the corresponding author upon reasonable request.

## Open Access

This article has been published open access under our Subscribe to Open programme, made possible through the support of our subscribing institutions, learn more here: https://portlandpress.com/pages/open_access_options_and_prices#conditional

## Abbreviations

FBS: fetal bovine serum
MHCII: major histocompatibility complex II
TLR: Toll-like receptor
iNOS: inducible nitric oxide synthase
