# Supplementary material for: Exosome-mediated modulation of macrophage polarization and inflammation in early Klebsiella pneumoniae lung infections
Source: Clin Sci (Lond). 2025 Dec 23;139(24):1691–707. doi: 10.1042/CS20256616 (PMC12794323; doi:10.1042/CS20256616)
Supplement: online supplementary material 1. [file cs-139-24-CS20256616-s001.pdf]

Supplementary Figures and legends:

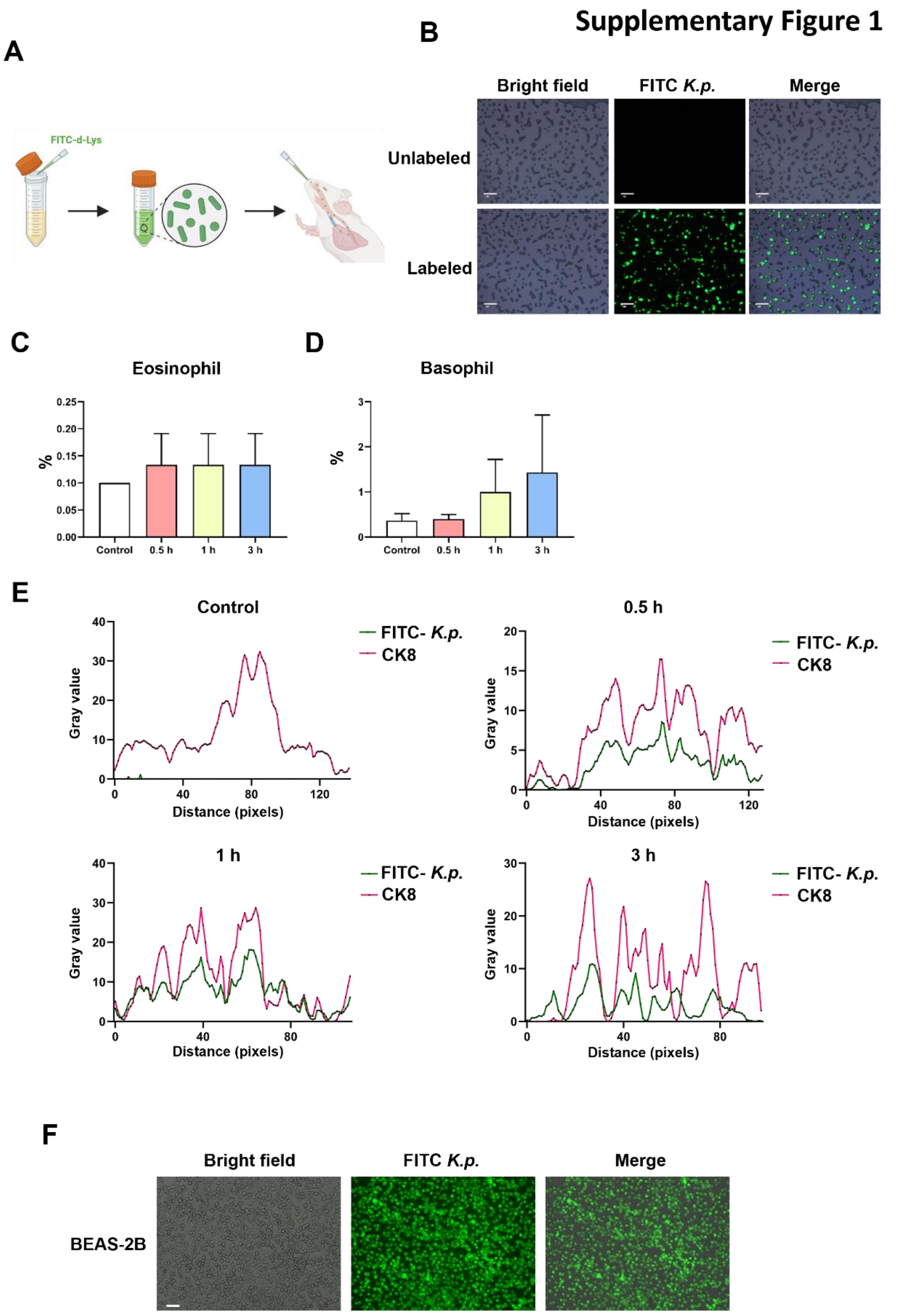

Supplementary Figure 1: Cellular and Tissue Response to FITC-Conjugated K. pneumoniae Infection

(A) Schematic illustration of the experimental procedure for labeling cells with FITC-conjugated K. pneumoniae and subsequent administration.

(B) Fluorescence microscopy images showing THP-1 cells labeled with FITC-conjugated K. pneumoniae (FITC K.p.). Bright field, FITC fluorescence, and merged images are displayed for both unlabeled and labeled cells.

(C-D) Quantitative analysis of immune cell populations in the lung tissue at various time points post-infection: (C) Eosinophils, (D) Basophils. Statistical significance is indicated: $^{**}P < 0.01$, $^{***}P < 0.001$.

(E) Gray value analysis along a defined line in the images, showing the distribution of FITC-K.p. and CK8 at different time points.

(F) Fluorescence microscopy images of BEAS-2B cells labeled with FITC-conjugated K. pneumoniae, showing bright field, FITC fluorescence, and merged images.

Supplementary Figure 2

A

Raman shift (cm^{-1})	Vibrational modes and/or assignments
855	1,4-glycosidic link stretching/Polysaccharide structure
1087	Skeletal C-C streteching/Lipids
1461	CH2 Bending/Lipids

B

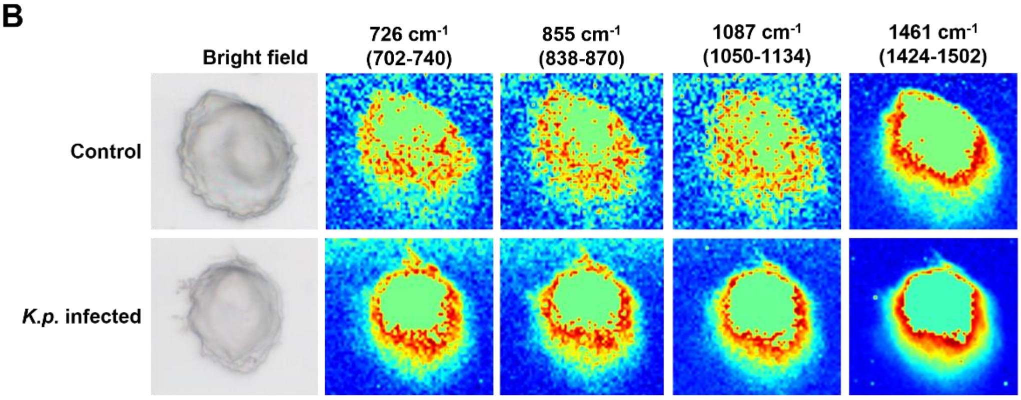

C

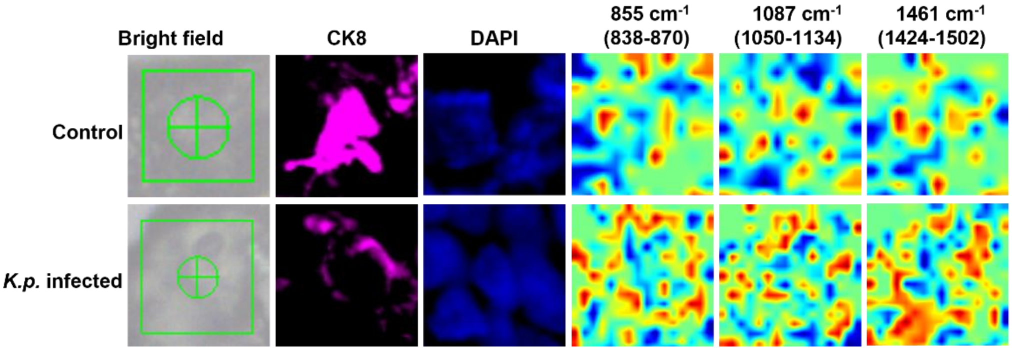

Supplementary Figure 2: Raman Spectroscopy Analysis of K. pneumoniae and Peptidoglycan

(A) Table listing Raman shifts and their corresponding vibrational modes or assignments.

(B) Raman spectral maps of control and K.p. infected samples at various Raman shifts, highlighting molecular composition changes.

(C) Bright field and Raman spectral images of two samples (#1 and #2), showing distribution of FITC-K.p. and corresponding Raman shifts.

Supplementary Figure 3

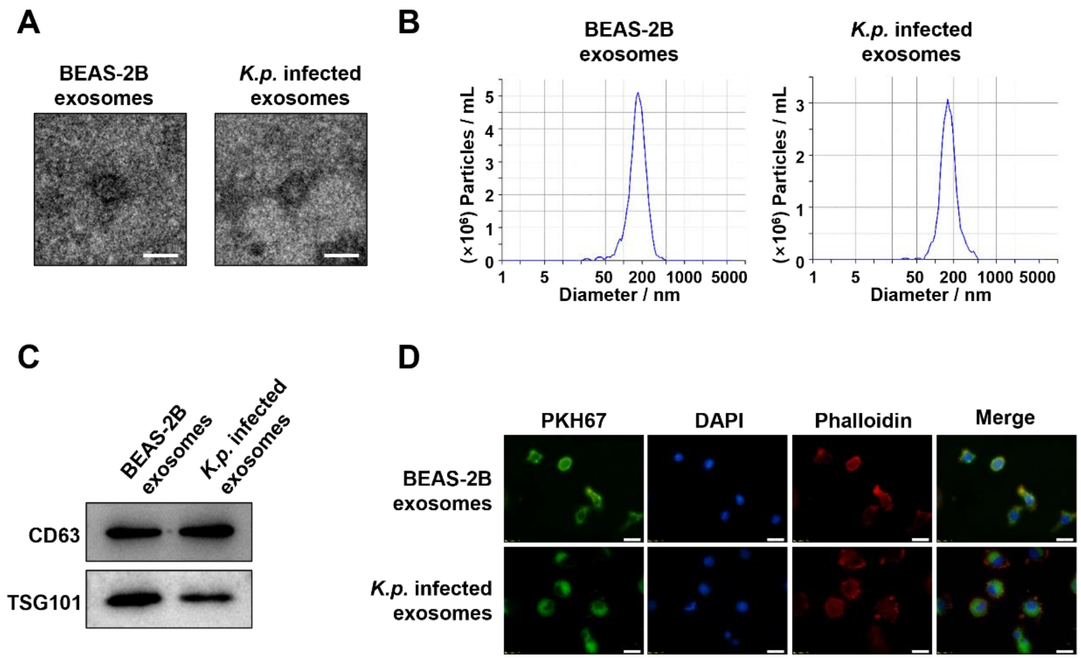

Supplementary Figure 3: The Characterization of Exosomes Secreted by BEAS-2B cell after K. pneumoniae Infection

(A) The representative images of exosomes secreted from BEAS-2B with or without K. pneumoniae Infection by electron microscopy.

(B) The particles isolated from indicated exosomes by nanoparticle tracking analysis.

(C) Representative Molecules Detected in indicated exosomes by Western Blot.

(D) The representative images of indicated exosomes by Immunofluorescence staining.
